# No Major Host Genetic Risk Factor Contributed to A(H1N1)2009 Influenza Severity

**DOI:** 10.1371/journal.pone.0135983

**Published:** 2015-09-17

**Authors:** Koldo Garcia-Etxebarria, María Alma Bracho, Juan Carlos Galán, Tomàs Pumarola, Jesús Castilla, Raúl Ortiz de Lejarazu, Mario Rodríguez-Dominguez, Inés Quintela, Núria Bonet, Marc Garcia-Garcerà, Angela Domínguez, Fernando González-Candelas, Francesc Calafell, CIBERESP Cases and Controls in Pandemic Influenza Working Group

**Affiliations:** 1 Institut de Biologia Evolutiva (CSIC-Universitat Pompeu Fabra), Barcelona, Spain; 2 Unidad Mixta de Investigación en Genómica y Salud CSISP-UVEG/Institut Cavanilles-Universitat de València, València, Spain; 3 CIBER de Epidemiologia y Salud Pública, Madrid, Spain; 4 Servicio de Microbiología Hospital Universitario Ramón y Cajal and Instituto Ramón y Cajal de Investigación Sanitaria (IRYCIS), Madrid, Spain; 5 Department of Microbiology, Hospital Universitari Vall d’Hebron (HUVH), Universitat Autònoma de Barcelona (UAB), Vall d’Hebron Institut de Recerca (VHIR), Barcelona, Spain; 6 Instituto de Salud Pública de Navarra, Pamplona, Spain; 7 Department of Microbiology and Immunology, Hospital Clinico Universitario, Valladolid, Spain; 8 Red Española de Investigación en Patología Infecciosa (REIPI), Madrid, Spain; 9 Grupo de Medicina Xenomica, Universidade de Santiago de Compostela, Centro Nacional de Genotipado—Plataforma de Recursos Biomoleculares y Bioinformaticos—Instituto de Salud Carlos III (CeGen-PRB2-ISCIII), Santiago de Compostela, Spain; 10 Departament de Salut Pública, Universitat de Barcelona, Barcelona, Spain; The University of Tokyo, JAPAN

## Abstract

While most patients affected by the influenza A(H1N1) pandemic experienced mild symptoms, a small fraction required hospitalization, often without concomitant factors that could explain such a severe course. We hypothesize that host genetic factors could contribute to aggravate the disease. To test this hypothesis, we compared the allele frequencies of 547,296 genome-wide single nucleotide polymorphisms (SNPs) between 49 severe and 107 mild confirmed influenza A cases, as well as against a general population sample of 549 individuals. When comparing severe vs. mild influenza A cases, only one SNP was close to the conventional p = 5×10^−8^. This SNP, rs28454025, sits in an intron of the *GSK233* gene, which is involved in a neural development, but seems not to have any connections with immunological or inflammatory functions. Indirectly, a previous association reported with *CD55* was replicated. Although sample sizes are low, we show that the statistical power in our design was sufficient to detect highly-penetrant, quasi-Mendelian genetic factors. Hence, and assuming that rs28454025 is likely to be a false positive, no major genetic factor was detected that could explain poor influenza A course.

## Introduction

In 2009, the influenza A(H1N1)2009 pandemic swept the globe. Some of its features caused concern, such as a higher mortality risk in infants and children than in seasonal influenza epidemics, and activity peaks out of the cold season. Although it followed a mild course in most patients, in others it was much more aggressive for then unknown reasons. In Spain, a collection of severe, hospitalized patients was compared to a series of controls (defined as confirmed but mild influenza cases that were treated ambulatorily), and a number of sociodemographic and health risk factors were identified [1]. Yet, 37.2% of the severe cases showed no clinical or sociodemographic risk factor for severe influenza.

Host genetic factors may be a contributor to influenza severity. Two genealogy studies in Utah and Iceland clearly demonstrated familial aggregation of the risk of influenza-associated death [2]. Albright et al. [3] compiled 4855 deaths due to influenza from a Utah database between 1904 and 2004, and observed that the relative risk for relatives dying of influenza was larger than for spouses. Thus, on top of the risk due to cohabitation and shared sociodemographic and environmental factors, the authors concluded that the risk of dying from influenza is heritable. On the contrary, in the 1918 influenza epidemic in Iceland [4], the 455 deaths showed no increased risk for the cases' relatives when compared to spouse's relatives. Notice that the sample in the Icelandic study was an order of magnitude smaller than the Utah sample, and its statistical power was presumably smaller.

Several studies have tackled human genetic variation in relation to influenza. A group of candidate genes includes the pro-inflammatory cytokines and chemokines. Morales-García et al.[5] compared Mexican influenza A patients and matching, co-inhabiting controls and found that single nucleotide polymorphisms (SNPs) in the *TNF* and *LTA* genes contributed to developing the disease, with an OR as high as 27 (95% CI 3–1248) for rs361525*AA in *TNF*. This allele was also found to be associated with influenza A in Greek patients, and in particular with those developing pneumonia (OR 3.74, 95% CI 1.06–13.25)[6]. However, it was not associated with fatal influenza (regardless of type, and accumulated over 10 influenza seasons) in 105 US children and young adults [7]. Another candidate gene is the interferon-inducible transmembrane protein *IFITM3*, which was shown to be essential against the influenza A virus in mice *in vivo*. A sample of 53 severe UK patients showed higher frequencies of the minor allele in an *IFITM3* SNP, namely rs12252, compared to population databases comprising 3,000–9,000 individuals [8]. Moreover, genetic variation around this SNP revealed the footprints of recent, positive natural selection in Europeans but not in Asians or Africans [8].

A genome-wide association study (GWAS) indicated the complement regulatory protein *CD55* as a possible candidate for severe A influenza [9]; indeed, genotype rs2564978*T/T in *CD55* showed an OR = 1.75 (P = 0.011) in 177 severe vs. 248 mild Chinese A(H1N1) cases [9]. Finally, a GWAS was also performed in 91 severe Mexican patients vs. 98 exposed but asymptomatic controls [10], by using an array designed for genes related with cardiovascular diseases. Four genome regions were identified as putative candidates to contribute to infectivity and/or severity; these regions contained genes for the immunoglobulin-related *FCGR2A*, the *RPA* interacting protein, and the complement-binding *C1QBP* protein, with ORs up to 2.63.

The goal of the present study is to explore the existence of major genetic determinants of influenza A(H1N1) severity by comparing the genotypes of a dense array of genomewide SNPs in 49 severe and 107 mild influenza patients from Spain, and in a general population sample of 549 individuals. To the best of our knowledge, this is the first attempt to analyze genetic factors associated to influenza infection in a population of European ancestry.

## Material and Methods

### Samples

Cases were defined as confirmed influenza A(H1N1)2009 patients who had to be admitted to a hospital. Genotypes were successfully obtained for 49 cases (27 from the Hospital Clínico de Valladolid, 17 from Hospital Ramón y Cajal, Madrid, and 5 from Hospital Virgen del Camino, Pamplona, all in Spain; 27 were women). Controls were also confirmed influenza patients with mild symptoms who received ambulatory assistance; they were recruited as part of the Spanish Influenza Case-Control Study cohort during the 2009–2010 first epidemic season of the new virus. 107 controls (56 women) were successfully genotyped: 28 originated from the PIDIRAC primary care influenza network in Catalonia, and 79 from the Red Centinela and Hospital Clínico in Valencia. Additionally, a sample from the general population was used in some comparisons. It consisted of 549 DNA samples from subjects that self-reported having at least two generations of ancestors born in Spain and without personal or familiar history of chronic diseases. These individuals were obtained from the CeGen-PRB2-ISCIII project, and consisted of unrelated healthy adult individuals collected from diverse geographic locations of Spain by Fundacion Publica Galega de Medicina Xenomica. Appropriate written informed consent was obtained from all participants in this study, which was approved by the Clinical Research Ethics Committee-Parc de Salut Mar, Barcelona.

### Influenza A virus detection and sample genotyping

Samples were divided into two aliquots to extract DNA and RNA separately. To extract DNA, samples were first incubated for an hour with a lysis buffer plus proteinase K to digest the cells membranes, followed by a standard phenol-chloroform purification and ethanol precipitation. RNA was extracted by incubating first with TRIzol reagent (phenol/guanidine isothiocyanate), then adding chloroform followed by a centrifugation step to recover the aqueous phase. We then incubated this with isopropanol for an hour followed by centrifugation and ethanol wash. Finally, the RNA pellet was diluted in RNase-free water. The WHO RT-PCR protocol [11] was followed to confirm infection by influenza A (H1N1)2009 virus.Samples were genotyped with the Affymetrix Axiom Genome-Wide Human CEU

Array, which contains 587,353 genome-wide SNPs and indels.

### Statistical analyses

Genotypes were called with the Affymetrix Power Tools 1.14.4 provided by the manufacturer; all samples were analyzed together to avoid array biases. A total of 156 samples (49 cases of influenza A and 107 mild cases) passed the quality controls suggested by the manufacturer. In addition, SNPs that failed in more than the 5% of samples were discarded, with 547,296 SNPs remaining. Allele frequencies have been deposited in GWAS Central as study HGVST1832 (http://www.gwascentral.org/study/HGVST1832) and in the figshare repository (http://dx.doi.org/10.6084/m9.figshare.1528227)

A genome-wide association study between severe and mild influenza cases was carried out using the program Plink 1.07 [12]. In addition, 549 population samples from Galicia (NW Spain) were used as controls. The significance threshold for multiple test correction was set at 5×10^−8^, as suggested by [13]

Population stratification was estimated with two approaches: identity-by-state among individuals was computed with PLINK 1.07 and subsequently plotted with multidimensional scaling. Independently, a Bayesian approach to population stratification with Admixture 1.22 [14] was also used. Graphical representations of results were made using the R language [15]

## Results

**Fig 1:**
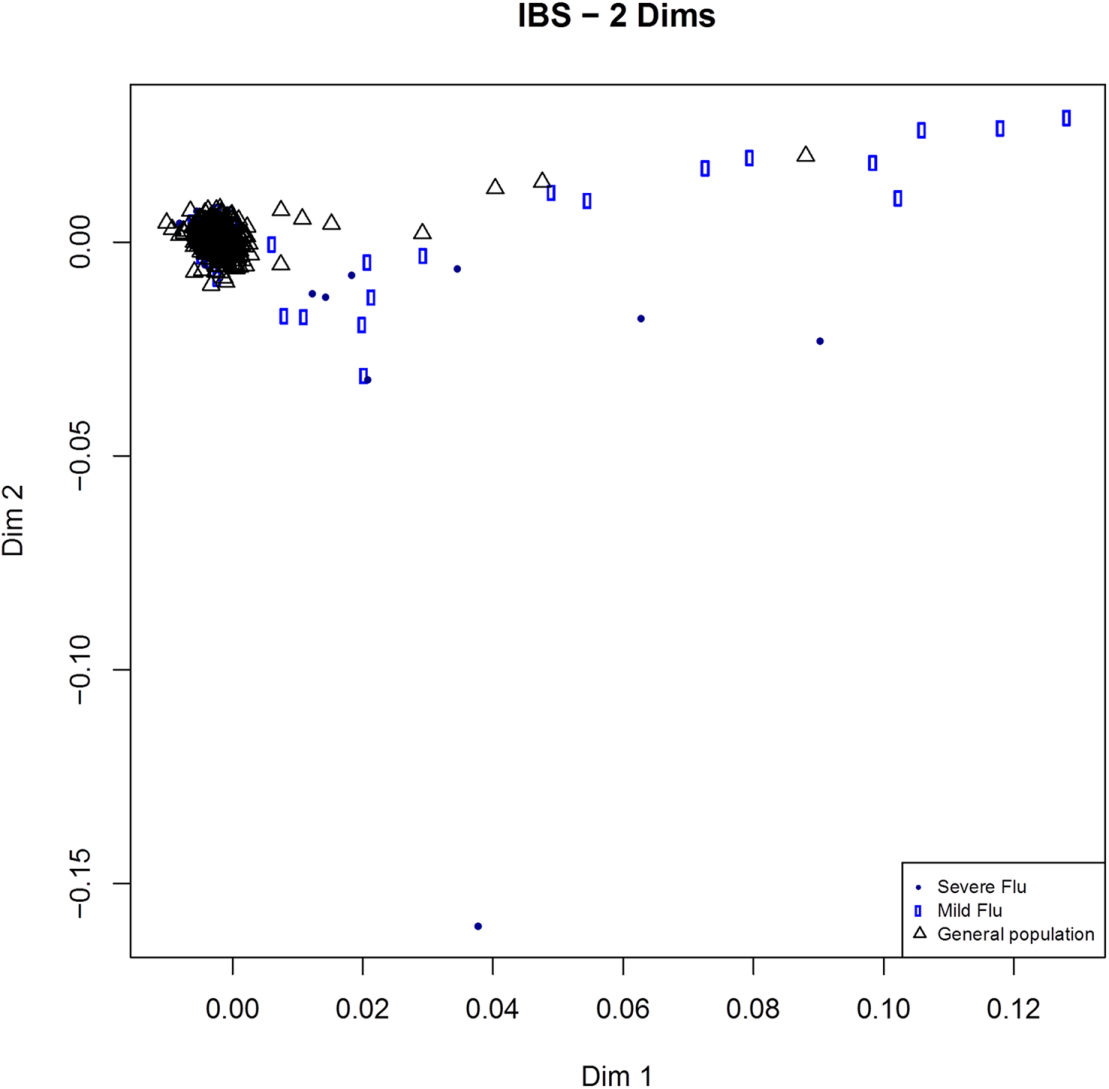
Multidimensional scaling based on identity by state distances among severe influenza cases, mild influenza cases, and a general population sample.

Population structure analysis using identity by state revealed a number of potential outliers: eight samples of severe influenza, 17 cases of mild influenza, and eight controls (Fig 1), which were removed from association analyses. ADMIXTURE analysis (S1 Fig) did not show any discernible pattern, and the optimal number of parental populations was K = 1, implying that cases and controls can be considered as having been sampled from the same population.

**Fig 2:**
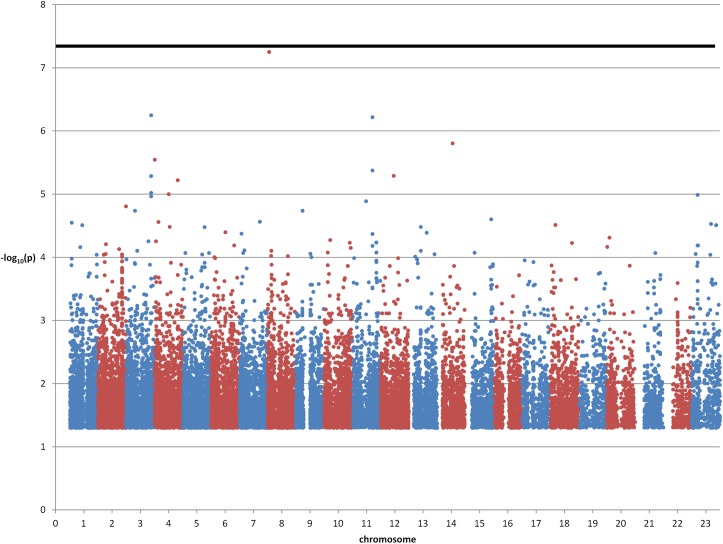
Manhattan plot of severe vs. mild influenza cases. Only p-values < 0.05 are shown. The thick horizontal line denotes p = 5×10^−8^.

The genome-wide association study between severe influenza A (41 cases) and mild influenza A (90 cases) revealed one SNP (rs28454025) slightly above multiple-testing significance level (unadjusted p = 5.595x10-8; the OR could not be computed since minor allele frequencies were 0.1579 in cases but 0 in controls) as shown in Fig 2. This SNP is located in an intron of the *SGK223* gene (homolog of rat pragma of Rnd2), which seems to regulate neurite outgrowth.

In addition, 10 SNPs had p < 10^−5^ (S1 Table). Three of these SNPs were in introns of *NAALADL2* (N-acetylated alpha-linked acidic dipeptidase-like 2), at a maximum distance of 67.9 Kb between each other. This gene has been associated with, among other diseases, systemic lupus erythematosus[16] and Kawasaki’s disease[17], both autoimmune disorders. Two other SNPs were in introns of *MAML2* (mastermind-like 2 (Drosophila)), a member of the Notch developmental pathway, and apparently unrelated to the immune function. We also found a 14-Kb block of 16 SNPs in the *PARD3B* gene (par-3 partitioning defective 3 homolog B (*C*. *elegans*), involved in neurodevelopment) with p < 10^−3^. The SNPs in this block had odd ratios between 2.1 and 2.9 and were ~1.4Mb downstream from *CTLA4*, a costimulatory molecule expressed by activated T cells that has been linked to a number of autoimmune diseases.

Next, we considered a subset of SNPs that were in candidate genes that had been previously associated with influenza A severity [8–10]. Unfortunately, none of the SNPs that had been previously associated were in the array we genotyped; therefore, we selected the SNPs that were within 100 Kb of the previously associated SNPs. Note that we applied a hypothesis-specific Bonferroni correction, taking into account the number of proxy SNPs (ranging from 2 to 52) we found for each previously associated SNP *IFITM3* is the gene that has been most consistently associated with response to influenza A [8]; and, in particular, the rs12252 SNP. Our array contained 27 SNPs in the vicinity, and only one, namely rs4131943, had a nominally significant association with influenza A severity (p = 2.1×10^−3^, OR = 2.60 95% CI 1.40–4.83); note that the hypothesis-specific number of tests was 27, and that the multiple testing correction would yield p = 0.0567. For *CD55* [9], within 100 Kb of the reported SNP, only two SNPs were available in our array, one of which, rs2564978, was significantly associated with influenza severity (p = 0.00638, OR = 7.11, 95% CI 1.4–36). Out of 11 SNPs in the vicinity of *FCGR2A* [10], only one had a nominally significant association with influenza severity (rs7551957, p = 0.0288), which did not survive Bonferroni correction. For the SNPs in the *RPAIN-C1QBP* region, out of 52 SNPs in the vicinity, only two had p<0.05, with the smallest value (p = 0.0203, corresponding to rs28447573, which was not the closest to any of the two previously associated SNPs) far from surviving Bonferroni correction. In summary, we indirectly replicated the association for *CD55*, that for *IFITM3* was close to significance, and we could not indirectly replicate the previous associations for *FCGR2A*, *RPAIN*, and *C1QBP*.

**Fig 3:**
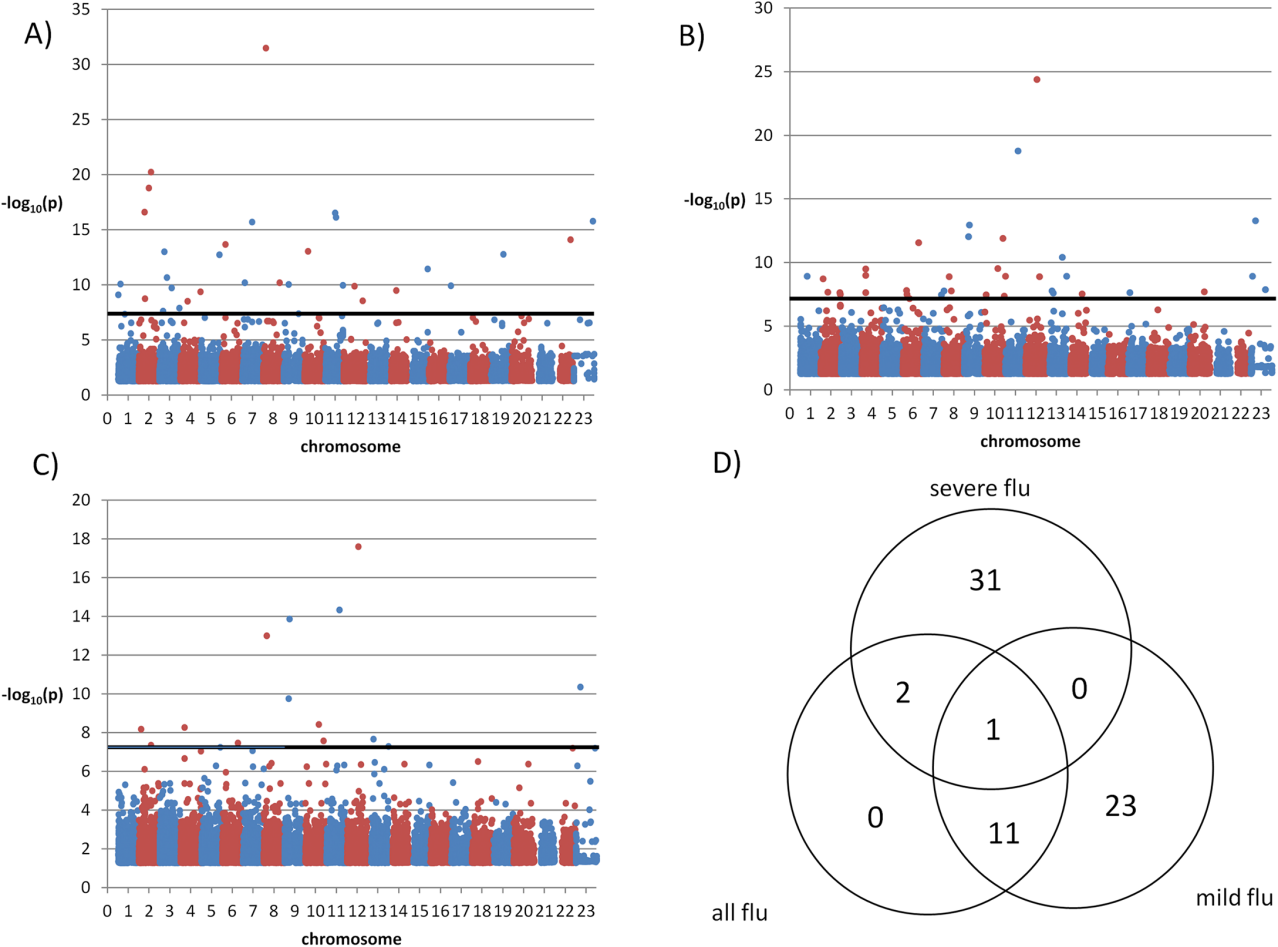
Manhattan plots of a general population sample vs. (A) severe influenza cases; (B) mild influenza cases and (C) all influenza cases. Only p-values < 0.05 are shown. (D): Venn diagram of SNPs with significant associations with p<5×10^−8^ when comparing severe influenza cases, mild influenza cases or all influenza cases to a general population sample.

We also compared the two sets of influenza patients to the general population (S2–S4 Tables) and, given the larger sample size of the control population, more SNPs yielded significant association tests (Fig 3). In the GWAS of severe influenza A against the general population 34 SNPs had p<5×10^−8^ and were in Hardy-Weinberg equilibrium (p > 0.05) (Fig 3A). In the analysis of mild influenza A against the general population 35 SNPs (Fig 3B) met the same conditions, and in the analysis of all influenza cases against the general population 14 SNPs had significant differences (Fig 3C). Out of these SNPs, 31 were specific of severe influenza A cases and 23 of mild influenza A cases (Fig 3D). The 34 SNPs detected in the comparison between severe influenza cases and the general population comprised 16 intergenic SNPs, 14 intronic SNPs, two synonymous SNPs, and two nonsynonymous SNPs: rs11551002 in gene *APLP1* (amyloid precursor-like protein 1, involved in neural function), and rs11216131 in *BUD13*, which participates in the regulation of serum lipid levels.

Next, we turned to the 49 genes that contained or were adjacent to the 34 SNPs with significant differences between severe cases and the general population (S2 Table). We screened their definitions in the Refseq database and their biological functions according to GO categories for involvement in immunity and inflammation, and the Genetic Association Database and CDC HuGe Published Literature for associations with autoimmune and inflammatory diseases. SNP rs1996377 was 3.09 Mb downstream from *DPP10*, which is associated with asthma [18]; and rs28447319 was 51.8 Kb upstream of *B4GALT1*, which is upregulated by proinflammatory TNFα[19].

## Discussion

In a GWAS for genetic contributors to the severity of influenza A infection, we did not find any SNP with p<5×10^−8^; the SNP with the lowest p-value (and one order of magnitude smaller than the next most associated SNP) was rs28454025, which lies in an intron of the *SGK223* gene. This gene encodes an enzyme that belongs to the tyrosine protein kinase family. A similar protein in rat binds to Rho family GTPase 2 (Rnd2) and regulates neurite outgrowth via activation of Ras homolog gene family, member A (RhoA) [20]. SNPs in SGK223 have only been putatively associated with carotid artery disease[21], an association that has not been subsequently replicated. The association between rs28454025 and influenza A severity seems, then, implausible.

Previous studies had produced a number of SNPs that were associated with influenza infection or severity. None of those were contained in the array we genotyped, but, by using as proxies SNPs in the genomic vicinity, we indirectly replicated the association with *CD55*, while *IFITM3* was close to statistical significance.

When we compared the severe influenza A cases with a much larger sample of the general population, 34 SNPs had significant allele frequency differences (p<5×10^−8^) However, the biological plausibility of most of these associations was tenuous (e.g. SNPs with distances >100 Kb from the closest gene, or in or near genes with biological functions unlikely to be related to inflammation or immunity).

Sample sizes in our study were low: 41 severe and 90 mild influenza A cases. Still, it has sufficient statistical power to rule out common genetic variants as highly penetrant contributors to poor prognosis in influenza A infection. The prevalence of severe influenza A among influenza A cases without obvious risk factors was estimated as 0.26% in a Spanish population[22]. We used this figure and Genetic Power Calculator [23] to estimate that our study had 0.415 power to detect a recessive variant with an allele frequency of 0.2 and an odds ratio of 25 with α = 5×10^−8^ and a dominant model; for a recessive model, power increased to 0.805. Admittedly, these parameters imply a penetrance sufficiently high so as that familial aggregation of non-cohabiting relatives may have been observed. In particular, in these conditions the relative risk for a sibling would be 1.562 in the dominant model and 2.404 in the recessive model.

In conclusion, our study did not detect what was powered to find, namely one or a few host genes with a major impact in poor influenza course. However, this does not rule out the presence of genes with a more limited contribution.

## Supporting Information

S1 Fig Population stratification analysis from K = 2 to K = 5. Top left, coefficient of variation for each K value.
(PPTX)

S1 Table SNPs with association p-values < 10^−5^ when comparing mild and severe influenza cases. Only rs16954376 remained significant after Bonferroni correction.
(XLSX)

S2 Table SNPs with significant association p-values (p < 5×10^−8^) when comparing severe influenza cases against a sample of the general population. (XLSX)

S3 Table SNPs with significant association p-values (p < 5×10^−8^) when comparing mild influenza cases against a sample of the general population. (XLSX)

S4 Table SNPs with significant association p-values (p < 5×10^−8^) when comparing all influenza cases against a sample of the general population. (XLSX)
