# Correction: No Major Host Genetic Risk Factor Contributed to A(H1N1)2009 Influenza Severity

**DOI:** 10.1371/journal.pone.0141661

**Published:** 2015-10-30

**Authors:** Koldo Garcia-Etxebarria, María Alma Bracho, Juan Carlos Galán, Tomàs Pumarola, Jesús Castilla, Raúl Ortiz de Lejarazu, Mario Rodríguez-Dominguez, Inés Quintela, Núria Bonet, Marc Garcia-Garcerà, Angela Domínguez, Fernando González-Candelas, Francesc Calafell, CIBERESP Cases, Controls in Pandemic Influenza Working Group

In the Funding section, the second grant number from the funder Instituto de Salud Carlos III (Madrid, Spain; www.isciii.es) is missing. The grant number is GR09/0021.
