# Supplementary figures and images for: No Major Host Genetic Risk Factor Contributed to A(H1N1)2009 Influenza Severity
Source: PLoS One. 2015 Sep 17;10(9):e0135983. doi: 10.1371/journal.pone.0135983 (PMC4574704; doi:10.1371/journal.pone.0135983)

## Slide 1
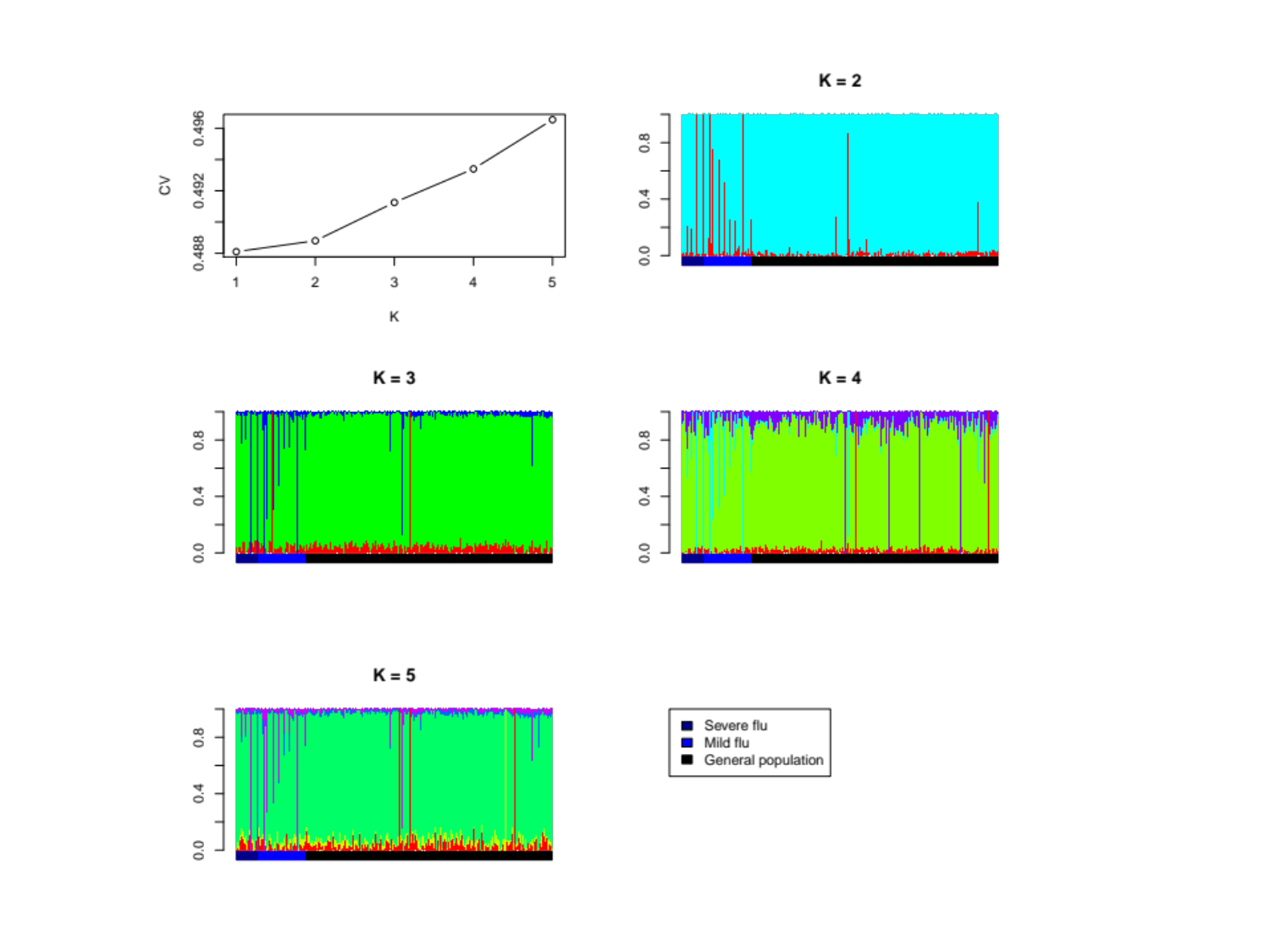

Supplement: S1 Fig — Top left, coefficient of variation for each K value. (PPTX) [file pone.0135983.s001.pptx]
